# Organocatalytic Oxidative Dehydrogenation of Dihydroarenes by Dioxygen Using 2,3-Dichloro-5,6-dicyano-benzoquinone (DDQ) and NaNO_2_

**DOI:** 10.3390/molecules13123236

**Published:** 2008-12-18

**Authors:** Wei Zhang, Hong Ma, Lipeng Zhou, Zhiqiang Sun, Zhongtian Du, Hong Miao, Jie Xu

**Affiliations:** 1 State Key Laboratory of Catalysis, Dalian Institute of Chemical Physics, Chinese Academy of Sciences, Dalian 116023, P. R. China; E-mail: zhangwei@dicp.ac.cn (W. Z), mahong@dicp.ac.cn (H. M.), szq@dicp.ac.cn (Z-Q. S.), duzhongtian@dicp.ac.cn (D-Z. T.), miaohong@dicp.ac.cn (H. M.); 2 Graduate University of the Chinese Academy of Sciences, Beijing 100049, P. R. China; 3 Department of Chemistry, Institute of Catalysis, Zhengzhou University, Zhengzhou 450001, P. R. China; E-mail: zhoulipeng@zzu.edu.cn (L-P. Z.)

**Keywords:** Oxidative dehydrogenation, DDQ, Dioxygen, Redox couples

## Abstract

The oxidative dehydrogenation of dihydroarenes catalyzed by 2,3-dichloro-5,6-dicyano-benzoquinone(DDQ) and NaNO_2_ with dioxygen is reported. The combination of DDQ and NaNO_2_ showed high efficiency and high selectivity, compared with other benzoquinones and anthraquinones, e.g., >99% conversion of 9,10-dihydroanthracene with 99% selectivity for anthracene can be obtained at 120 °C under 1.3 MPa O_2_ for 8 h. Excellent results were achieved in the oxidative dehydrogenation of variety of dihydroarenes.

## Introduction

The dehydrogenation of hydrocarbons to various alkenes and aromatic olefins is an important project in modern chemical manufacture and scientific research [1, 2]. A very attractive approach is oxidative dehydrogenation. Oxidants such as dioxygen, halogens, sulfur compounds, etc., could accept hydrogen and thus the thermodynamics of the dehydrogenation process shifts to the right side. These exothermic oxidation reactions also provide necessary process heat to compensate for the endothermic dehydrogenation and thus dehydrogenation can be successfully operated under moderate conditions [3]. On the other hand, the selectivity is difficult to control in the presence of oxidants, and byproducts are thus generated. Recently, noble-metal catalysts such as Ru(OH)_x_/Al_2_O_3_ and Ru(TMP)(O)_2_ have been found to readily catalyze oxidative dehydrogenation reactions with high selectivity [4, 5], but unfortunately the need for noble metals makes such a dehydrogenation process uneconomic. There is strong incentive for developing new cheaper oxidative dehydrogenation catalysts which simultaneously employ dioxygen as oxidizing agent.

High-potential quinones such as DDQ are usually used as hydrogen acceptors in many types of reactions such as dehydrogenation of hydrocarbons and benzylic alcohols, oxidation of alcohols and allylic ethers, and direct cross-dehydrogenative-coupling reactions, etc [6,7,8,9]. Unfortunately, quinones are often used with stoichiometric amount because they are converted into quinols after accepting hydrogen and can not be recycled to the initial state. In order to solve the above problem, efforts have been made to regenerate the quinones. Several reagents such as Mn(OAc)_3_, HClO_4_, HIO_4_, and HNO_3_ showed efficiency in converting quinols to quinones [10, 11]. But in most cases these reagents were still required in stoichiometric or in excess amounts.

**Scheme 1:**
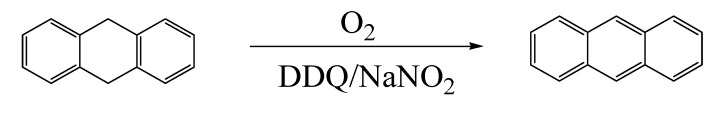
Catalytic oxidative dehydrogenation of 9,10-dihydroanthracene to anthracene.

In a previous study at our laboratory, anthraquinones and NHPI were coupled to form an efficient organocatalytic system in the aerobic oxidation of hydrocarbons under moderate reaction conditions. The redox transformation between anthraquinones and anthraquinols can be cycled by NHPI/PINO [12,13,14,15]. Recently, we have designed a novel DDQ/NaNO_2_ catalytic system for dehydrogenation of 9,10-dihydroanthracene (Scheme 1) [16]. In the present work, benzoquinones and anthraquinones combined with NaNO_2_ were studied in terms of activity and selectivity. Further, the detailed operation conditions including catalyst loading, reaction time, and temperature for DDQ/NaNO_2_ were optimized. Moreover, its application in the oxidative dehydrogenation of variety of dihydroarenes was investigated. At last the redox catalytic cycle was proposed. The redox couples quinone/quinol and NO_2_/NO were expected to create a catalytic cycle in the presence of O_2_ in the dehydrogenation. This novel organocatalytic system offers new thoughts for design of highly selective catalyst for oxidative dehydrogenation of aromatic hydrocarbons.

## Results and Discussion

### Comparison of different benzoquinones and anthraquinones combined with NaNO_2_

**Figure 1:**
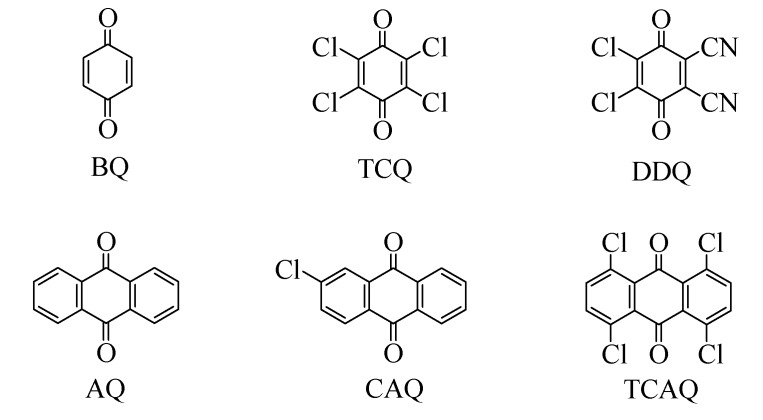
The studied benzoquinones and anthraquinones.

BQ: benzoquinone; TCQ: 2,3,5,6-tetrachloro-1,4-benzoquinone; AQ: anthra-quinone; CAQ: 2-chloroanthraquinone; TCAQ: 1,4,5,8-tetrachloroanthraquinone

**Figure 2:**
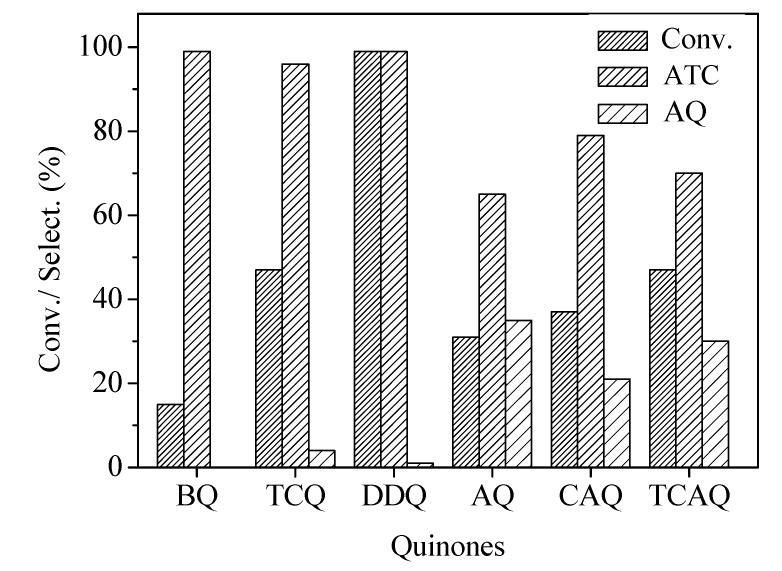
The oxidative dehydrogenation of 9,10-dihydroanthracene with different quinones and NaNO_2_^a^.

^a^ Reaction conditions: 10 mL toluene, 0.5 mmol 9,10-dihydroanthracene, 0.05 mmol quinone, 0.05 mmol NaNO_2_, 120 °C, 1.3 MPa O_2_, 8 h. When using AQ/NaNO_2_, the amount of by-product AQ is the difference between the GC measurement value and the loading amount in AQ/NaNO_2_. ATC: anthracene

The effect of a variety of benzoquinones and anthraquinones (Figure 1) including BQ, TCQ, DDQ, AQ, CAQ, and TCAQ combined with NaNO_2_ in the oxidative dehydrogenation of 9,10-dihydroanthracene was explored (Figure 2).

The results showed that both benzoquinones and anthraquinones exhibited considerable activity. It was notable that DDQ/NaNO_2_ showed high catalytic efficiency. The conversion of 9,10-dihydroanthracene was >99% and selectivity to anthracene was 99%. Besides DDQ/NaNO_2_, TCQ/NaNO_2_ also showed good results. The selectivity to anthracene was 96% with 47% conversion. Comparably, BQ/NaNO_2_ was low active but high selective. The conversion only reached 15% and the selectivity to 9,10-dihydroanthracene was >99%. Traces of AQ were detected as the sole by-product by GC-MS measurement. The activity of benzoquinones varied following the order: DDQ>TCQ>BQ. These activity results coincide with the reduction potential order of benzoquinones [17,18,19]. The electron-withdrawing group can increase the reduction potential and thus the dehydrogenation ability increases. In case of different anthraquinones (AQ, CAQ, and TCAQ), the results were not satisfactory, as both anthracene and considerable amounts of AQ were produced. The occurred oxidative side-reaction suggested anthraquinones have catalytic ability in catalyzing aerobic oxidation of 9,10-dihydroanthracene to AQ. On the basis of these results, it could be concluded that DDQ combined with NaNO_2_ was the highest active and selective among the quinones investigated.

### Optimization of the catalyst loading amounts, reaction time, and temperature for DDQ/NaNO_2_

Performing as an excellent oxidative dehydrogenation catalyst, DDQ combined with NaNO_2_ was further studied under detailed conditions. The results of oxidative dehydrogenation with different catalyst loading amount are listed in Table 1. The loading amounts of DDQ and NaNO_2_ were equal and increased simultaneously from 1 mol% to 10 mol%. Obviously, the conversion is low at lower catalyst loading (1 mol%, 2 mol%, and 3 mol%), and then increased to >99% at 5 mol% and 10 mol%. So 5% DDQ and 5% NaNO_2_ are selected as the optimum loading amounts. Furthermore, we examined the efficiency of air. With 5% DDQ and 5% NaNO_2_, it only gave 64% conversion and >99% selectivity to anthracene under 1.3 MPa air. By increasing the catalyst loading amount to 10% DDQ and 10% NaNO_2_, the conversion was increased to >99% with >99% selectivity to anthracene, similar to that under 1.3 MPa O_2_. The results indicated that oxidative dehydrogenation can perform under air, but the efficiency is lower than that under O_2_.

**Table 1:** Effect of catalyst loading amount on the oxidative dehydrogenation of 9,10-dihydroanthracene to anthracene ^a^.

Entry	Catalyst loading (mol%)		Conversion (%)	Products and selectivity (%)	
	DDQ	NaNO_2_		ATC	AQ
1	1	1	19	>99	-
2	2	2	35	>99	-
3	3	3	56	>99	-
4	5	5	>99	99	1
5	10	10	>99	99	1
6 ^b^	5	5	64	>99	-
7 ^b^	10	10	>99	>99	-

^a^ Under the same reaction conditions as described in Figure 2; ^b^ 1.3 MPa air.

**Table 2:** Effect of reaction time on the catalytic oxidative dehydrogenation of 9,10-dihydroanthracene to anthracene ^a^.

Entry	Catalyst loading (mol%)		Time (h)	Conv. (%)	Products and selectivity(%)	
	DDQ	NaNO_2_			ATC	AQ
1	5	5	1	42	>99	-
2	5	5	3	47	>99	-
3	5	5	5	54	>99	-
4	5	5	8	>99	99	1
5	5	0	5	10	>99	-
6	0	5	5	8	>99	-
7	0	0	5	3	>99	-
8^ b^	0	0	5	n.d.	-	-

^a^ Under the same reaction conditions as described in Figure 2; ^b^ under N_2_ atmosphere.

In order to further study the process, the influence of reaction time on the catalytic performance of DDQ/NaNO_2_ was investigated. As illustrated in Table 2, the conversion of 9,10-dihydroanthracene increased very rapidly in the initial 1 h (entry 1). When the reaction time was up to 8 h, the conversion of 9,10-dihydroanthracene reached >99%. In the whole reaction process, the selectivity for anthracene was extremely high, especially in the initial 1-5 h, and no oxidative by-products were detected (entries 1-3). To identify the characteristics of each components of DDQ/NaNO_2_ and function of O_2_, further study was carried. The reactions were run for 5 h, avoiding the disturbance of further slight oxidation occurring at long reaction times. It was observed that 54% of 9,10-dihydroanthracene was dehydrogenated with DDQ/NaNO_2_ (entry 3). When 5 mol% DDQ was used individually, the conversion was decreased dramatically from 54% to 10% (entry 5). It suggested NaNO_2_ can promote DDQ dehydrogenate 9,10-dihydroanthracene. Only using 5 mol% NaNO_2_, dehydrogenation reaction can proceed to a limited extent with 8% conversion (entry 6). Moreover, without DDQ/NaNO_2_, only a small amount of anthracene was produced (entry 7). This indicated that 9,10-dihydroanthracene is difficult to dehydrogenate with NaNO_2_ or O_2_. In this system, NaNO_2_ readily decomposes to NO. NO can be rapidly oxidized to NO_2_ in the presence of O_2_, and the generated NO_2_ can oxidize 2,3-dichloro-5,6-dicyano-1,4-hydroquinone (DDQ-H_2_) to DDQ. Similarly, the quick oxidation of NO to NO_2_ by O_2_ was observed in aerobic oxidation of alcohols using TEMPO/Br_2_/NaNO_2_ catalyst [20], and also found in CH_4_ oxidation with Pd/C/NaNO_2_/BQ [21]. It cannot proceed without the organocatalytic system in the absence of O_2_ (entry 8). This proved the indispensability of O_2_.

**Figure 3:**
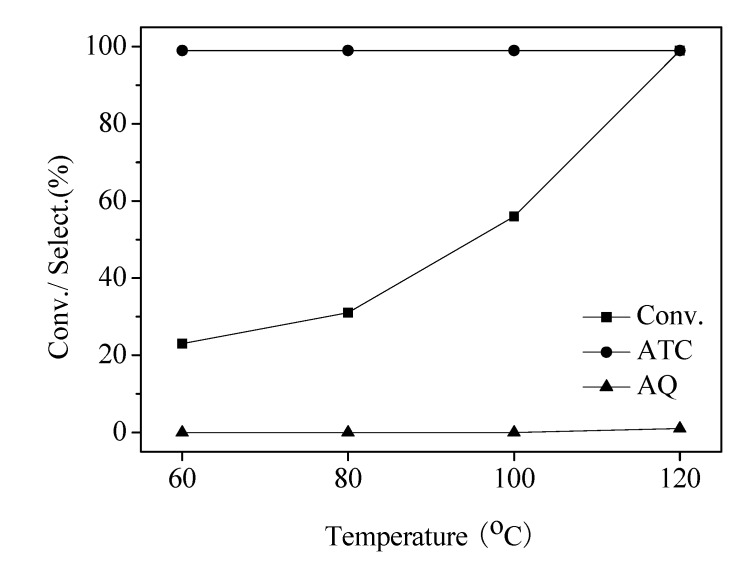
Effect of reaction temperature on the oxidative dehydrogenation of 9,10-dihydroanthracene ^a^.

^a^ 0.025 mmol quinone, 0.025 mmol NaNO_2_; other conditions were the same as described in Figure 2.

The effect of temperature on the rate of reaction was examined with DDQ (5 mol%) and NaNO_2_ (5 mol%) for 8 h (Figure 3). It can be seen that elevating the reaction temperature from 60 ^o^C to 120 ^o^C barely affected the selectivity to anthracene, which remained at high values (>99% at 60 ^o^C-100 ^o^C, and 99% at 120 ^o^C). On the other hand, the conversion of 9,10-dihydroanthracene was significantly increased when enhancing the reaction temperature. At 120 ^o^C, the conversion of 9,10-dihydroanthracene achieved >99%. The results indicated that the optimized temperature for oxidative dehydrogenation with DDQ/NaNO_2_ was 120 ^o^C.

### Oxidative dehydrogenation of variety of dihydroarenes with DDQ/NaNO_2_

To explore the application scope of this organocatalytic system, oxidative dehydrogenation of variety of dihydroarenes including 9,10-dihydroanthracene, 1,4-cyclohexadiene, acenaphthene, 9,10-dihydrophenanthrene, 1,2-dihydronaphthalene, and iminodibenzyl by DDQ/NaNO_2_ were investigated (Table 3).

**Table 3:** Oxidative dehydrogenation of different dihydroarenes with DDQ/NaNO_2_^a^.

Entry	Substrates	Conversion (%)	Product and selectivity/yield (%)
1	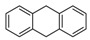	>99	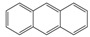 99/99
2	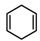	>99	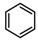 >99/99
3	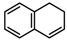	91	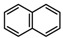 >99/91
4	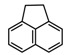	77	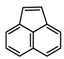 >99/77
5	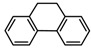	68	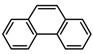 >99/68
6^b^	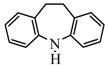	32	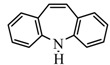 74/24

^a^ Reaction conditions: 0.5 mmol substrate; other conditions were the same as described in Figure 2; ^b^ the main by-product cannot be confirmed.

This organocatalytic system exhibited high selectivity. In the cases of 9,10-dihydroanthracene, 1,4-cyclohexadiene, and 1,2-dihydronaphthalene, yields to anthracene, benzene, naphthalene were 99%, 99%, and 91%, respectively. For other substrates, moderate yields (around 70%) were achieved under the same reaction conditions (entries 4, 5). When iminodibenzyl was reacted, it only gave 32% conversion with 74% selectivity to iminostilbene. It is worthwhile to note that Thummel *et al*. had examined the DDQ-induced dehydrogenation rates in the aromatization of 1,4-dihydro-benzocycloalkenes and 1,4-dihydronaphthocycloalkenes. They provided evidence to support that a positively charged intermediate was involved in an initial rate-limiting hydride transfer to DDQ. The intermediate carbonium ion would be destabilized by electron-withdrawing groups and stabilized by the electron-donating groups, which causes different hydride losing rates [22]. Here the phenomenon that iminodibenzyl showed low activity was consisting with their findings. It may probably because the electron-withdrawing effect of a N atom destabilized the intermediate carbonium ion, and thus the activity of iminodibenzyl was reduced.

### The proposed catalytic cycle

Based on the above results, the functions of the DDQ and NaNO_2_ are as follows. (1) DDQ can dehydrogenate 9,10-dihydroanthracene to anthracene. (2) NaNO_2_ is suggested to act as a convenient nitrogen oxide source, and to readily decompose to NO. NO will rapidly react with O_2_ to form NO_2_. The released NO_2_ can abstract hydrogen from 9,10-dihydro-anthracene to produce anthracene. Since it is difficult to dehydrogenate 9,10-dihydroanthracene with NO_2_, the reaction is kept at low conversion. In the absence of O_2_, NO can not be oxidized to NO_2_, and thus dehydrogenation can not occur. (3) O_2_ may be responsible for the recovery of NO_2_ from NO, and NO_2_ will abstract hydrogen from DDQ-H_2_ to generate DDQ, and thus the dehydrogenation by DDQ can proceed. In our previous work, the suggestion was proven by two additional experiments [16]. When 0.5 mmol DDQ and 0.5 mmol 9,10-dihydroanthracene were stirred at 120 ^o^C for 8 h without O_2_, it was found that 99% of 9,10-dihydroanthracene was converted to anthracene and considerable amounts of DDQ-H_2_ were detected by HPLC measurements. This demonstrated that DDQ can stoichiometrically dehydrogenate 9,10-dihydroanthracene to anthracene. Then 0.5 mmol NaNO_2_ and 0.5 mmol DDQ-H_2_ were mixed and stirred under 1.3 MPa O_2_, DDQ-H_2_ was fully oxidized to DDQ. It revealed that in the presence of O_2_, NO can be oxidized to NO_2_, and NO_2_ can readily oxidize DDQ-H_2_ to DDQ.

**Scheme 2:**
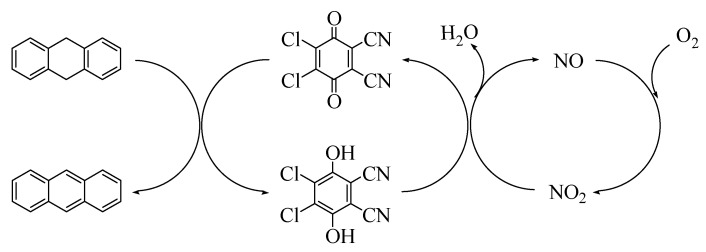
The proposed catalytic cycle of DDQ/NaNO_2_ for oxidative dehydrogenation of 9,10-dihydroanthracene to anthracene.

A tentative mechanism for the catalytic cycle is proposed in Scheme 2. The substrate 9,10-dihydroanthracene is readily dehydrogenated by DDQ to produce anthracene. After abstracting hydrogen, DDQ is reduced to DDQ-H_2_. DDQ-H_2_ is re-oxidized to DDQ again by NO_2 _ generated *in-situ*, and NO is produced. In the presence of O_2_, NO is readily oxidized to NO_2_. Thus the dehydrogenation can proceed. The net reaction is: 9,10-dihydroanthracene reacted with O_2_ to form anthracene and H_2_O.

## Conclusions

In conclusion, a quinone-based organocatalytic system was developed for the oxidative dehydrogenation of 9,10-dihydroanthracene with dioxygen under mild conditions. Compared with other quinones, DDQ combined with NaNO_2_ showed high efficiency. It gave >99% conversion of 9,10-dihydroanthracene with 99% selectivity for anthracene at 120 °C under 1.3 MPa O_2_ for 8 h. The organocatalytic system was also efficient for the oxidative dehydrogenation of variety of dihydroarenes. Two redox couples – DDQ/DDQ-H_2_ and NO_2_/NO – were combined to create a catalytic cycle in the presence of O_2_. DDQ/NaNO_2_ is highly effective and economical. It may be one of the substitutions for the conventional oxidative dehydrogenation catalyst.

## Experimental

### General

BQ (99%), TCQ (99%), DDQ(98%), AQ (99%), CAQ (99%), TCAQ (99%), 9,10-dihydro-anthracene (98%), anthracene (99%), 1,4-cyclohexadiene (97%), acenaphthene (99%), 9,10-dihydro-phenanthrene (95%), 1,2-dihydronaphthalene (>98%), and iminodibenzyl (97%) were purchased from J&K Chemical Ltd. NaNO_2_ (99%) and toluene (99.5%) were purchased from Tianjin Kermel Chemical Reagent Development Center.

### Oxidation reaction and products analysis

The typical catalytic reaction was performed in a 50 mL stainless steel autoclave equipped with a magnetic stirrer. Toluene (10 mL), 9,10-dihydroanthracene (0.5 mmol), quinine (0.05 mmol), and NaNO_2_ (0.05 mmol) were placed in the autoclave. After the desired temperature was reached, O_2_ was pressurized (ca. 1.3 MPa) into the reactor and the pressure was kept constant by supplying dioxygen during the reaction. The oxidation products were identified by Agilent 6890N GC/5973 MS detector and quantitated by Agilent 4890D GC equipped with FID detector.
